# Does environmental uncertainty of enterprises aggravate the accrual anomaly in the stock market? Evidence from China

**DOI:** 10.3389/fpsyg.2022.1006957

**Published:** 2022-10-11

**Authors:** Shuya Hu, Shengnian Wang

**Affiliations:** Department of Accounting, School of Economics and Management, Shihezi University, Shihezi, Xinjiang, China

**Keywords:** environmental uncertainty, accrual anomaly, investor irrationality, economic man, state-owned enterprise, corporate governance

## Abstract

Enterprises do not exist independently of the external environment, so uncertainty affects their earnings volatility and exacerbates the information asymmetry between internal and external stakeholders. As a major manifestation of capital market mispricing, the accrual anomaly is caused by investors’ functional fixation on total surplus under information asymmetry. Against this backdrop, taking A-share listed companies in China from 2007 to 2019 as our research objects, this study explores the impact of environmental uncertainty on the accrual anomaly based on the information asymmetry and investor irrationality perspective. We find that environmental uncertainty enterprises facing exacerbates the accrual anomaly in the Chinese stock market, and internal control quality, state ownership and the media coverage will affect this impact. Furthermore, this study shows that there are three factors playing the mediating role in the effect, accounting information quality, investment growth and the investor attention. The results show that environmental uncertainty exacerbates the accrual anomaly driven by information manipulation, empire building and investor irrationality. Improving investor irrationality behavior and restraining the self-interest behavior of managers can help alleviate the mispricing of accruals caused by information asymmetry in psychology.

## Introduction

The efficient markets hypothesis (EMH) holds that securities prices reflect all available capital market information at any time, thus ruling out the possibility of arbitrage behaviors successfully yielding excess returns (Fama, 1970). In recent years, various capital market anomalies have occurred frequently, raising doubts about this hypothesis. By constructing a portfolio, Sloan (1996) documented that the accrual component of earnings is less persistent than the cash flow component and concludes that investors fail to appreciate the differing properties of accruals and cash flows comprehensively. He was the first scholar to confirm the existence of the accrual anomaly. Since then, a number of scholars have confirmed that the accrual anomaly exists widely in the capital market (Francis et al., 2005; Pincus et al., 2007)—it is not an exception but a common phenomenon in global capital markets. As a representative emerging capital market, the Chinese capital market suffers from information asymmetry, which leads to a more pronounced presence of the accrual anomaly. Particularly, the stock market is dominated by irrational investors in China. Therefore, it is necessary to determine what factors influence this accrual anomaly in China.

Enterprises cannot exist independently of the external environment, which inevitably affects their operating activities. Under the influence of changing economic conditions and rising geopolitical frictions, every enterprise is affected by different degrees of environmental uncertainty. On the one hand, high environmental uncertainty makes strategic planning more difficult and increases the future operational risk, resulting in dramatic performance fluctuations. And the burst of uncertainty information about performance can distract investors’ limited attention, resulting in investors’ inability to judge risk information and reducing their decision-making efficiency. On the other hand, it also aggravate the degree of information asymmetry. This makes it difficult to supervise executive behaviors, offering management more opportunities to hide their private behavior and increasing the difficulty faced by external investors in monitoring them (Ghosh and Olsen, 2009). Currently, the Chinese economy has entered a stage of high-quality development. With the improvement of China’s economic status, Chinese enterprises face an array of domestic and foreign impacts. Enterprises in various industries are operating in a more turbulent internal and external environment than ever before. High environmental uncertainty makes enterprises, investors, regulators and other subjects face higher information asymmetry, which complicates external supervision and internal auditing, providing fertile conditions for management to build empires and manipulate earnings (Habib et al., 2010). Meanwhile, high uncertainty also increases the cost of information screening for market participants, which is detrimental to their decision-making activities.

Research on the causes of the accrual anomaly has featured four main hypotheses: the model setting defect hypothesis (Hirshleifer et al., 2005), the limited arbitrage hypothesis (Collins et al., 2003), the investment growth hypothesis (Fairfield and Yohn, 2003; Zhang, 2007) and the earnings persistence hypothesis (Hong, 2001). It is widely accepted that the accrual anomaly is a manifestation of market failure under information asymmetry. Based on this perspective, environmental uncertainty is one important factor that may aggravate the accrual anomaly in the Chinese capital market and reduce capital market pricing efficiency.

**Figure 1:**
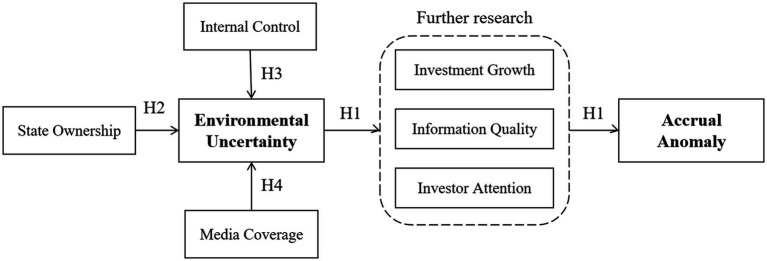
The logical framework diagrams.

Against this backdrop, this study explores the impact of environmental uncertainty of enterprises on the accrual anomaly and tests the mechanism in the relationship, in the special institutional context and current economic environment of China. The data consist of annual firm-level data on Chinese listed companies from the China Stock Market & Accounting Research (CSMAR) database from 2007 to 2019. And the logical framework diagrams are showen in Figure 1. First, using multiple regression models, we initially verify the existence of the accrual anomaly in the Chinese capital market, finding that this anomaly is not an exception but rather a common phenomenon in global capital markets. Meanwhile, by constructing environmental uncertainty indicators at the macro, meso and micro levels, we test the relationship between environmental uncertainty and the accrual anomaly, finding that environmental uncertainty exacerbates the accrual anomaly in the Chinese capital market. Second, to explore whether there is heterogeneity in the relationship in different situations, the study constructs a grouped regression. Chinese state-owned enterprises play a dual role in commercial and public welfare, so we group firms based on their ownership and conclude that state ownership exacerbates the impact of environmental uncertainty on the accrual anomaly. And the quality of internal control reflects the level of corporate governance, so we group firms based on this indicator and find that better internal controls can mitigate the impact of environmental uncertainty on the accrual anomaly. Meanwhile, as one of the mediums of information transmission, there are still some disputes about whether media reports can play an effective role, so we also group firms based on this indicator and find that more media reports can exacerbates the impact of environmental uncertainty on the accrual anomaly. Third, there are many factors affecting the accrual anomaly. To determine the particularity of its behavior in the Chinese stock market, the study further clarifies how it is affected by environmental uncertainty. On the one hand, based on rational economic man theory, we propose that a lack of monitoring triggered by environmental uncertainty increases management’s desire for empire building, while performance volatility due to environmental uncertainty increases management’s surplus manipulation ideas. Environmental uncertainty breeds self-interested behavior of management, whether information manipulation or empire building, is a significant factor in exacerbating the accrual anomaly. On the other hand, based on investor irrationality in psychology, volatility risk to companies due to environmental uncertainty can distract investors from their limited focus, leading to that they unable to identify the authenticity of information and lock in total earnings psychologically. Thus, this study takes accounting information quality, enterprise investment growth and investor attention as the intermediary variables and tests for the mediating effect between environmental uncertainty and the accrual anomaly, finding that environmental uncertainty exacerbates the anomaly by reducing accounting information quality, increasing corporate investment growth and diverting investor attention. In addition, the paper conducts multiple robustness tests by altering the sample selection criteria, substituting variables, implementing a placebo test and measuring environmental uncertainty from multiple perspectives to support the results reported above. All of these results are in line with expectations.

This study contributes to the literature on three fronts. First, it adds to the literature on the economic consequences of environmental uncertainty. The exiting literature around it focuses on corporate governance (Kim and Yasuda, 2021), corporate strategy (Mu et al., 2020), performance (Lestari, 2017) and external stakeholders (Chow et al., 2018). However, while related work has examined its impact on stock prices, little evidence has been provided that environmental uncertainty affects capital market anomalies. This study argues that environmental uncertainty is one of the factors that affect the information asymmetry between enterprises and investors and cannot be ignored in explorations of capital market anomalies. Second, it enriches the literature on the accrual anomaly in stock market. The research on the accrual anomaly addresses mainly earnings management (Bansal and Ali, 2021), enterprise growth (Doukakis and Papanastasopoulos, 2014), arbitrage activities (Sullivan and Zhang, 2017), investor irrationality (Beer et al., 2018), and analyst coverage (Gordon et al., 2014). There is little evidence on the accrual anomaly from perspective of change in the enterprise environment. This study explores the problem from the perspective of internal and external environmental factors, providing new ideas for subsequent research. Third, it extends the literature on the mechanism whereby the external environment affects capital market pricing efficiency. The literature on the accrual anomaly focuses on direct effects, and few works have studied the specific mechanism of action. By examining the effects of environmental uncertainty on the accrual anomaly from accounting information quality, investor attention and enterprise investment growth view, the mechanism tests support the investment growth hypothesis (Zhang, 2007) and the earnings persistence hypothesis (Hirshleifer et al., 2012; Bansal and Ali, 2021). And the results also explain clearly how environmental uncertainty affects the accrual anomaly and expand the research on indirect effect and direct effect. Fourth, it also supports the literature on negative effects of media coverage. The existing research on media coverage is controversial, mainly focusing on mitigating information asymmetry (Bushee et al., 2010) and aggravating information asymmetry (Core et al., 2008). Our study runs grouped regressions with differences in media coverage and finds that environmental uncertainty has a more significant effect on accrual anomalies in samples with high media coverage, thus supporting the noise effect of media coverage.

At the same time, the conclusion of the article generates practical insights for us. First, by conducting heterogeneity analysis from three perspectives and exploring the impact of environmental uncertainty in different contexts on accrual anomalies, we find that the special attributes of state-owned enterprises and media coverage are important factors that exacerbate the relationship between environmental uncertainty and the accrual anomaly, while the quality of internal control can effectively mitigate the relationship between them, providing empirical evidence on how enterprises can effectively respond to environmental uncertainty. Second, the mechanism of environmental uncertainty affecting the accrual anomaly is explored from several perspectives. And it is found that environmental uncertainty exacerbates the anomaly by reducing the quality of accounting information, increasing investment growth, and reducing investor attention, which provides practical guidance for alleviating the accrual anomaly in the Chinese capital market. Third, our research also pointed out some current situations of China’s capital market, such as the rampant self-interest behavior of management, serious irrational behavior of investors, and the failure of the media supervision mechanism. Therefore, the conclusion can also provide reference for relevant regulatory authorities to formulate regulatory measures.

The rest of the paper proceeds as follows. Section 2 introduces the institutional background and explains our rationale for selecting a sample from China. Section 3 analyzes the related theories and develops the hypotheses. Section 4 presents the data sources and defines the variables. Section 5 discusses our empirical strategy and the research design. Section 6 reports and analyzes the results. Section 7 conducts further exploration. Section 8 concludes.

## Background

We set our study sample in Chinese A-share listed companies, on the one hand, because of the specificity of the Chinese sample, mainly in terms of emerging market characteristics, economic environment and the composition of market participants. First, as a representative of emerging markets, China’s capital market started late but developed rapidly, leading to the emergence of the drawbacks of uneven capital market development, and is an economy that cannot be ignored when studying emerging markets (Du et al., 2020). However, the unbalanced development of the capital market stands out in its daily operation, for example, in the high frequency of market anomalies, which has stunted economic growth. Second, as the second largest economy, China’s rapid economic growth has brought more opportunities and challenges for its companies, but it is also an important factor in exacerbating the uncertainty faced by domestic companies. Emerging markets face an environment of greater uncertainty, volatility and ripple effects (Meyer and Peng, 2016). Foreign companies pour in to China divide the market, and global economic downturn increases crisis they are trapped in. Both of them exacerbate environmental uncertainty for Chinese companies. Third, different from the market structure of the developed capital market, China’s capital market is dominated by minority investors, which are more susceptible to environmental interference and irrational behavior, presenting the unique market characteristics (Venky et al., 2019). Therefore, in the increasingly complex international situation of the Chinese market, the irrational behavior of investors in response to the uncertainty of the environment has become an important factor limiting their financial development.

On the other hand, we set the study environment in China because of the important impact of China’s problems on the world economies, mainly in promoting the rapid growth of emerging markets and facilitating international economic and financial cooperation among various countries. First, as a representative of emerging economies, China’s economy is currently in a period of social and economic transformation. Exploring China’s problems under this background will help promote the rapid growth of emerging markets (Wang, 2019). Uncertainty in the transition economy brings unpredictable changes to companies, which not only interferes with management risk management, but also enhances investors’ risk perceptions. Therefore, exploring the relationship between environmental uncertainty and accrual anomalies in this context not only identifies risks in emerging markets, but also is a driving force behind the rapid growth of emerging economies around the world. Second, the stable growth of China’s economy helps to promote the economic and financial cooperation among various countries internationally. In recent years, China’s capital market has been expanding its opening to the outside world and has gradually stepped onto the big stage of the world economy and finance (Johansson and Ljungwall, 2009; Wang, 2014). Exploring the response of companies there to the risk of uncertainty helps to achieve the stability of the capital market and is the basis for the promotion of economic cooperation among various countries. Above all, we conduct research on China, which is of great theoretical and practical significance.

## Hypothesis development

### Environmental uncertainty and the accrual anomaly

Driven by global economic integration, enterprise relationships are becoming increasingly close; no enterprise can exist independently, and firm performance can vary with the external environment. Therefore, unpredictable behavior on the part of external subjects, such as customers, suppliers, competitors, media, supervisory and management departments, may impose highly uncertain risks on individual enterprises (Satyanarayan, 1999). Both enterprise activity and investor decision-making in capital markets are affected by various environmental uncertainties, reflected mainly in the following three aspects. First, from the perspective of the psychology, environmental uncertainty can affect investors’ emotions, distract their attention and interfere with their cognition. With the interference of the environment and cognitive biases, investors may find it difficult to distinguish between accrual persistence and cash flow persistence. This exacerbates the market’s functional fixation on earnings persistence, leading to the more serious accrual anomaly. Second, environmental uncertainty exacerbates the degree of information asymmetry among market participants and increases the difficulty of predicting and supervising management behavior (Baum et al., 2010), which provides favorable conditions for management to hide decision-making errors and cover up poor management. And everyone in the behavioral finance perspective is a rational broker and has a self-interest need. So the uncertainty offers fertile conditions for managers to seek private benefits and build empires. Finally, environmental uncertainty increases both the amplitude and frequency of corporate performance volatility, which may prompt management to recognize surpluses that have yet to be realized to smooth performance (Merchant, 1990). This misleads investors to overestimate the persistence of corporate accruals (Kang, 2010). Environmental uncertainty not only exacerbates the information asymmetry between internal and external stakeholders, but also interferes with the limited cognition of irrational investors, which worsens the accrual anomaly. In view of all of the above, we formulate the first hypothesis as follows:

*H1:* Environmental uncertainty exacerbates the accrual anomaly in Chinese stock markets.

### Ownership, environmental uncertainty and the accrual anomaly

As the backbone of China’s national economic development, state-owned enterprises are one of the important driving forces of China’s rapid economic development. In China, state-owned enterprises play a dual role in commercial and public welfare and have significant responsibility for social and policy objectives (Lin and Tan, 1999). They also aim to achieve economic growth and help improve the effect of macroeconomic regulation and control. Therefore, the government offers state-owned enterprises preferential investment policy to stimulate economic growth while also obligating state-owned enterprises to shoulder all manner of extra costs (Xu et al., 2010). At the same time, the government helps state-owned enterprises obtain loans from the banking system to ease their financing constraints under environmental uncertainty, aiming to provide financial support to facilitate investment growth. Meanwhile, as agents of state-owned equity, state-owned executives have a strong incentive to make physical investments to achieve political goals (Shen and Lin, 2016). Under high environmental uncertainty, the degree of information asymmetry is more serious, which may serve as a rare opportunity for management over-investment behavior. Under the impact of environmental uncertainty, compared with state-owned enterprises, non-state-owned enterprises are subject to more serious financing and budget constraints; meanwhile, their ability to take risks is weak. All of these factors make management in non-state-owned enterprises more cautious in investment decision-making. These enterprises may have to reduce investment or postpone some activities to deal with environmental uncertainty. Since accruals are regarded as a component of growth in net operating assets, an increase in business investment activities inevitably leads to an increase in accruals. Investment growth follows the law of diminishing marginal returns on asset growth, and any increase in business investment activities conforms to this law, resulting in lower returns on future stocks. Therefore, compared with non-state-owned enterprises, state-owned enterprises enjoy greater resource endowments that play a key role in aggravating the impact of environmental uncertainty on the accrual anomaly. In view of the above, we formulate the third hypothesis:

*H2:* Environmental uncertainty has a more significant impact on the accrual anomaly in state-owned than in non-state-owned enterprises.

### Internal control, environmental uncertainty and the accrual anomaly

Enterprises with higher environmental uncertainty suffer more serious earnings volatility. They also face a higher risk of strategic failure. In such situations, management, as the core of enterprise operations, faces the double pressure of maintaining operating performance and addressing future environmental uncertainty, so it has an incentive to manipulate corporate earnings to smooth income (Davila et al., 2006). Internal control systems, as an important institutional arrangement in internal corporate governance mechanisms and an essential component of modern management, are designed to facilitate and accelerate companies’ achievement of objectives such as performance, disclosure and legitimacy goals in the whole operation process (Saarni, 2012). Thus, the internal control system plays an indispensable role in corporate governance, and high-quality internal control signals good corporate governance (Cormier et al., 2013). On the one hand, high-quality internal control can improve the stability of business operations and weaken the internal and external impacts of environmental uncertainty on enterprises. At the same time, it can effectively reduce opportunistic management behavior and improve corporate governance. Therefore, high-quality internal control enables enterprises to develop smoothly under high levels of environmental uncertainty. On the other hand, because it guarantees the regulation of disclosure behaviors and the provision of high-quality accounting information, high-quality internal control can alleviate the degree of information asymmetry between investors and enterprises and provide an effective basis for investors to evaluate the intrinsic value of the company and make investment decisions (Doyle et al., 2007). Therefore, with high-quality internal control, enterprises are better prepared to resist environmental uncertainty shocks (Mu et al., 2020). As a vital supervision system, the internal control system can weaken the information asymmetry between enterprises and investors, reduce management agency costs, and alleviate the impact of environmental uncertainty corresponding to the accrual anomaly. Considering all of the above, we formulate the second hypothesis:

*H3:* High-quality internal control can alleviate the impact of environmental uncertainty on the accrual anomaly.

### Media coverage, environmental uncertainty and the accrual anomaly

The news media is the window to the world for people, but with a two-sided nature. On the one hand, from the perspective of communication science, the media has become an important medium to alleviate information asymmetry by disseminating facts in the media and using objective and truthful judgment standards to process and disseminate information. But that is not the case. Excessive media coverage may not generate incremental information, but rather become an important distraction from investor attention, creating a noise effect that leads to biased investor decisions (Core et al., 2008). And the media is not always neutral, in order to pursue economic benefits, they may also provide readers with reports that tend to be entertaining or interesting (Bertrand and Mullainathan, 2003), which can trigger optimistic or pessimistic expectations (Tetlock, 2007). Therefore, media coverage not only amplifies the environmental uncertainty faced by companies, but also leads to the dissemination of biased information (Jia et al., 2020). On the other hand, based on a monitoring perspective, the media plays a governance function and becomes a complementary mechanism independent of the judiciary, the legislature and the executive. However, excessive media reports will lead to increased pressure on management, especially for companies facing high environmental uncertainty. So management under media pressure will be more inclined to tend to resort to surplus manipulation to meet analyst or investor expectations. Therefore, media report may not have a governance effect, but rather amplify corporate risks and disrupt stakeholder decisions. Considering all of the above, we formulate the second hypothesis:

*H4:* Media coverage can exacerbate the impact of environmental uncertainty on the accrual anomaly.

## Research design

### Data

The sample is from Chinese A-share listed companies and spans 2007 to 2019. We conduct preliminary processing of the sample as follows: (i) we remove financial and insurance firms to ensure comparability between firms in the sample; (ii) we remove special treatment (ST) firms to ensure the sample comprises firms without abnormal performance; (iii) we remove firms with missing and outlier observations. The sample selection process results in a final sample of 16,281. To alleviate the influence of extreme values on parameter estimation, observations with values below 1% or above 99% for continuous variables are winsorized. All data were obtained from CSMAR, WIND, DIB, CNRDS and the National Bureau of Statistics of China. The financial data are from CSMAR, the market risk coefficient is from WIND, the internal control quality data are from the DIB database, the media coverage data from CNRDS, and the Banker’s Macroeconomic Confidence Index is from the National Bureau of Statistics of China. Then, we process the data and graphs by using Stata 16.

### Variables

#### Cumulative abnormal return

In China, listed companies disclose their annual reports from January 1 to April 30 each year. Therefore, following prior studies, we adopt the market adjustment method to calculate firms’ cumulative abnormal return (Chen et al., 2013; *CAR*). A complete financial year is considered to be 1 May of the current year (year t) to 30 April of the following year (year t + 1). Then, based on the daily cumulative excess return of individual stocks, we adjust the figure to obtain the cumulative excess return of year t.


(1) $$AR_{i,d}=R_{i,d}-R_{p,d}$$

(2) $$CAR_{i,t}=\sum AR_{i,d}$$

where *AR_i,d_* is the excess return of stock i on the d trading day, *R_i,d_* is the return rate of stock i on the d day, and *R_p,d_* is the comprehensive daily market return rate on the d day considering the reinvestment of cash dividends, *CAR_i,t_* is the annual cumulative excess return.

#### Accrual earnings (*ACCR*) and cash flow earnings (*CASH*)

Following Collins et al. (2003) research, we use the cash flow statement method to calculate accrual earnings (*ACCR*) and cash flow earnings (*CASH*). The concrete calculating methods are as follows. First, we calculate CASH by dividing the cash flow from operations at the end of year t by the total assets at the end of year t. Second, net profit at the end of year t is subtracted from cash flow from operation. Third, we divide the result by the total assets at the end of year t and then calculate *ACCR*.

#### Environmental uncertainty

To fully explore the impact of environmental uncertainty on the accrual anomaly, we build three methods to measure environmental uncertainty from the micro, intermediate and macro perspectives. The respective indicators are *EU1*, *EU2*, and *EU3*.

*EU1*: The coefficient of variation is a statistic of the degree of variation of each observation, and it allows comparisons across industries of different sizes. Thus, we measure *EU1* as the coefficient of variation in sales income, following prior studies (Ghosh and Olsen, 2009). First, we calculate the standard deviation of sales revenue in the past 5 years from year t. Second, we standardize the standard deviation of sales revenue by industry, aiming to eliminate the impact of industry characteristics on the results, and from this step, we obtain the micro-level environmental uncertainty (*EU1*). Equation (3) is specified as follows:


(3) $$\mathrm{SALE}=\phi_{0}+\phi_{1}\mathrm{ACCPER}+\delta$$

*EU2*: Using the Zhi and Hull (2012) method, we use environmental complexity as an alternative measure of environmental uncertainty at the intermediate level. We take the three-level industry classification code revised by the China Securities Regulatory Commission (CSRC) in 2012 as the classification standard. Then, we take the natural logarithm of the statistics of competitors in the same industry, which we define as *EU2*. The greater the value is, the higher the environmental uncertainty.

*EU3*: As one of the indicators to describe macro environmental conditions, the Bankers’ Macroeconomic Confidence Index not only shows banks’ prediction of future economic trends but also represents their confidence in the macro economy. Therefore, we adopt the index as a substitute for macro environmental uncertainty (*EU3*). In facilitate interpretation and keep the results consistent with the other measurements, we use the opposite of the annual mean of the index (*EU3*) to measure environmental uncertainty. The greater the value is, the higher the environmental uncertainty.

#### Ownership (*SOE*)

According to who currently controls the enterprise, we divide enterprises into two ownership categories: state-owned and non-state-owned (Cull and Xu, 2004). *SOE* is a dummy variable that equals 1 if the actual controller of the enterprise is the central or a local government and 0 otherwise.

#### Internal control quality (*IC*)

The quality of internal control is essential for listed companies. Drawing on existing research results, we adopt the internal control index issued by DIB to measure it. *IC* is defined as a dummy variable that equals 1 if the internal control index is more than the mean in the same year and 0 otherwise.

#### Media coverage (*MC*)

Drawing on existing literature, we use media coverage of listed companies in the news as a measure of media attention. The media data provided by CNRDS database not only contains news reports from 20 mainstream online financial media and more than 400 other websites, but also covers news data from more than 600 important newspaper media, which is extensive and representative. Therefore, we sum the data from online and paper reports to calculate the total media attention. *MC* is defined as a dummy variable that equals 1 if the total media report is more than the mean in the same year and 0 otherwise.

#### Control variables

Based on existing research on the accrual anomaly (Christina et al., 2006; Zhang and Wilson, 2018), we select enterprise scale (*SIZE*), the earnings–price ratio (*EP*), the asset–liability ratio (*LEVEL*) and enterprise risk (*BETA*) as control variables. Meanwhile, to control for the impact of other non-observed factors in this study, we add an annual dummy variable (*ACCPER*) and an industry dummy variable (*INDUSTRY*) to the model.

The variable definitions are shown in Table 1.

**Table 1:** Variable definitions.

Variable name	Variable definitions
*CAR*	Based on the daily cumulative excess return of individual stocks, it is adjusted to the cumulative excess return of year t.
*EU1*	Coefficient of variation in sales income.
*EU2*	The natural logarithm of the statistics of competitors in the same industry.
*EU3*	The opposite number of the Banker’s Macroeconomic Confidence Index.
*ACCR*	The net profit minus cash flow from operating activities divided by total assets.
*CASH*	Cash flow from operation divide by the total assets at the end of the year t.
*SIZE*	The natural logarithm of the total assets of an enterprise at the end of year t.
*LEVEL*	The total liabilities are divided by the total assets at the end of year t.
*EP*	Net profit at the end of year t divided by stock market price.
*BETA*	The risk coefficient of market risk at the end of the year t.
*IC*	A dummy variable that equals 1 if the internal control index is more than the mean in the same year and 0 otherwise.
*SOE*	A dummy variable that equals 1 if the actual controller of the enterprise is the central or a local government and 0 otherwise.
*MC*	A dummy variable that equals 1 if the total media report is more than the mean in the same year and 0 otherwise.

This table describes the definitions of key variables employed in the study.

## Research design

To test the hypothesis, we use ordinary least squares regression analysis; the equations are described in Eqs. (4) and (5).


(4) $$\begin{array}{c} \begin{array}{l} CAR_{i,t+1}=\alpha_{0}+\alpha_{1}\;ACCR_{i,t}+\alpha_{2}\;CASH_{i,t}+\alpha_{3}\;SIZE_{i,t}\\ +\alpha_{4}\;\mathrm{LEVE}L_{i,t}+\alpha_{5}\;EP_{i,t}+\alpha_{6}\;\mathrm{BET}A_{i,t}+\mathrm{ACCPER}\\ +\mathrm{INDUSTRY}+\varepsilon_{i,t} \end{array}\\ \; \end{array}$$

where *CAR_i,t + 1_* is the cumulative excess return in the following year and the current year is year t. We adjust the daily cumulative excess return of individual stocks to the cumulative excess return of year t + 1. *ACCR_i,t_* is accounting earnings excluding the cash flow component, and *CASH_i,t_* is the accounting earnings from cash flow. *SIZE_i,t_* is the log total market value of equity at the end of year t. *LEVEL_i,t_* is the ratio of total liabilities to total market value of equity in year t. *EP_i,t_* is the net profit at the end of year t divided by the stock market price. *BETA_i,t_* is the risk coefficient of market risk at the end of year t. ε_i,t_ is the error term.

Equation (4) tests whether there is a significant accrual anomaly in the Chinese stock market during the sample period. To control for potential omitted variables, we add industry and time fixed effects to all regressions. If investors can identify the difference between accrual earnings and cash flow earnings and do not overestimate the persistence of accrual earnings, their investment decision-making is rational. However, if investors cannot distinguish the difference between the two components, they will overestimate the persistence of accrual earnings. Therefore, based on current conditions and the analysis above, we predict that there is a negative correlation between accrual earnings and cumulative excess returns in the next period, that is, that the coefficient on *ACCR_i,t_* is negative, α1 < 0.


(5) $$\begin{array}{c} \begin{array}{l} CAR_{i,t+1}=\beta_{0}+\beta_{1}\;EU_{i,t}+\beta_{2}\;ACCR_{i,t}+\beta_{3}\;EU_{i,t}\\ {}*ACCR_{i,t}+\beta_{4}\;CASH_{i,t}+\beta_{5}\;SIZE_{i,t}+\beta_{6}\;\mathrm{LEVE}L_{i,t}\\ +\beta_{7}\;EP_{i,t}+\beta_{8}\mathrm{BET}A_{i,t}+\mathrm{ACCPER}+\mathrm{INDUSTRY}+\varepsilon_{i,t} \end{array}\\ \; \end{array}$$

where *EU_i,t_* is measured with the three indicators described above at the micro, medium and macro levels: *EU1, EU2* and *EU3*. EU_i,t_*ACCR_i,t_ is the result for different *EU_i,t_* indicators multiplied by *ACCR_i,t_*.

Equation (5) tests the four hypotheses developed in this paper, where *β_3_* tests the impact of environmental uncertainty on the accrual anomaly. To control for potential omitted variables, we add industry and time fixed effects to all regressions. We predict a negative coefficient on EU_i,t_*ACCR_i,t_ if environmental uncertainty aggravates the accrual anomaly, *β_3_* < 0. Next, to conduct further research, on the basis of hypotheses 2, 3 and 4, we test the correlation between environmental uncertainty and the accrual anomaly in different situations. We conduct three group tests on subsamples based on the quality of internal control and firm ownership, named Group 1, Group 2, and Group 3. If ownership affects the correlation between the environmental uncertainty and the accrual anomaly, we predict that a negative coefficient on EU_i,t_*ACCR_i,t_ for state-owned enterprises and a positive or nonsignificant coefficient for non-state-owned enterprises in the subsample regression for Group 1. If internal control quality alleviates the correlation between environmental uncertainty and the accrual anomaly, we predict a negative coefficient on EU_i,t_*ACCR_i,t_ under poor internal control and a positive or nonsignificant coefficient under higher quality in the subsample regression for Group 2. If media coverage affects the correlation between the environmental uncertainty and the accrual anomaly, we predict that a negative coefficient on EU_i,t_*ACCR_i,t_ under high coverage and a positive or nonsignificant coefficient under poor coverage in the subsample regression for Group 3.

## Empirical results

### Descriptive statistics

Table 2 reports the descriptive statistical results for each major variable. The mean *CAR* is 0.145, the median is 0.046, and the standard deviation is 0.435, which indicates that there are significant differences in cumulative excess returns among different enterprises. Although there are three methods to measure environmental uncertainty, the standard deviation of each measurement is large. This shows that different enterprises face extremely different environmental uncertainties, which provides empirical support for the appropriateness of research on the correlation between environmental uncertainty and the accrual anomaly. The average fraction of accrual earnings (*ACCR*) is 0.005, and the average fraction of cash flow earnings is 0.055. Clearly, accrual earnings are lower than cash flow earnings. And the median of *ACCR* is −0.001, and it indicates that more than half of the samples have negative accruals. The mean of *SOE* is 0.519. it shows that more 52% of the sample are state-owned enterprises, indicating that state-owned enterprises play an important role in Chinese economic development. Then the findings for the control variables align with results from prior studies, which shows that there is no obvious errors in the estimation process.

**Table 2:** Descriptive statistics of major variables.

Variable	N	Mean	Std. dev.	Min	Median	Max
*CAR*	16281	0.145	0.435	−0.639	0.047	1.711
*EU1*	16281	1.739	1.958	0.000	1.076	10.60
*EU2*	16281	6.138	0.916	3.434	6.332	7.296
*EU3*	16281	57.69	12.53	35.60	56.63	75.28
*ACCR*	16281	0.005	0.064	−0.157	-0.001	0.215
*CASH*	16281	0.055	0.071	−0.150	0.053	0.253
*SIZE*	16281	22.40	1.303	20.06	22.22	26.39
*LEVEL*	16281	0.453	0.195	0.063	0.456	0.859
*EP*	16281	0.039	0.034	0.001	0.030	0.183
*BETA*	16281	1.147	0.264	0.492	1.145	1.944
*IC*	16281	0.500	0.500	0.000	1.000	1.000
*SOE*	16281	0.519	0.500	0.000	1.000	1.000
*MC*	16281	0.500	0.500	0.000	0.000	1.000

The table reports descriptive statistics of key variables over the sample period, 2007–2019.

### Correlation analysis

Table 3 reports the correlations among variables. The correlation coefficients between the cumulative excess return and the accrual earnings are −0.007, which preliminarily verify that there is an accrual anomaly in the Chinese capital market. All of the coefficients for the correlations between any two variables are lower than 0.8 except that for the correlation between the coefficients on accrual earnings and cash flow earnings. Since both the accrual and cash flow components are based on the total accounting surplus under the cash flow statement method, the correlation between the two coefficients is high, and the VIF test will be performed in the subsequent test to exclude the multicollinearity problem in the model. This result meets the econometric requirement of non-collinearity. That is, the variables in the regression equation do not exhibit a serious multiple collinearity problem.

**Table 3:** Correlation coefficient between variables.

	*CAR*	*EU1*	*ACCR*	*CASH*	*SIZE*	*LEVEL*	*EP*	*BETA*
*CAR*	1	−0.028***	−0.007	0.017**	−0.151***	−0.054***	0.017**	−0.102***
*EU1*	−0.017**	1	0.022***	−0.044***	0.225***	0.167***	0.055***	0.011
*ACCR*	−0.007	0.030***	1	−0.817***	−0.031***	0.019**	0.062***	0.057***
*CASH*	−0.001	−0.036***	−0.834***	1	−0.007	−0.173***	0.206***	−0.142***
*SIZE*	−0.124***	0.161***	−0.038***	−0.011	1	0.494***	0.426***	−0.044***
*LEVEL*	−0.040***	0.111***	0.028***	−0.181***	0.494***	1	0.132***	0.005
*EP*	−0.005	0.081***	0.064***	0.149***	0.463***	0.182***	1	−0.091***
*BETA*	−0.082***	0.015*	0.045***	−0.125***	−0.076***	−0.002	−0.074***	1

*, **, *** Denote significance at the 10, 5, and 1% levels, respectively. Multiple measurements of environment uncertainty will product multivariable multicollinearity. Meanwhile, whether it is macroscopic, middle, or microcosmic environment uncertainty, the impact of uncertainty on firm will be reflected on the enterprise performance in the end. So we only use one of the methods *EU1* to conduct correlation analysis between variables.

### Main results

Table 4 reports the results for our main analysis. The regression of Eq. (4) is used to test whether there is an accrual anomaly in the Chinese capital market, and the results are reported in column (1). We find that the coefficient on *ACCR_i,t_* is negative and significant at 1%, *α_1_* = −0.709, which shows that there is a negative correlation between accrual earnings and cumulative excess returns in the next period. The results indicate that investors over-evaluate accrual earnings in the Chinese stock market, that is, there was a significant accrual anomaly in the capital market during the sample period. The regression of Eq. (5) is used to test the correlation between environmental uncertainty and the accrual anomaly, where *β_3_* reflects the impact of the former on the latter. As commented earlier, we use three methods to measure environmental uncertainty in the regression, and the main results are shown in columns (2)–(4). We find that the coefficient on EU_i,t_*ACCR_i,t_ is −0.131 in column (2), which is negative and significant at 1%. We also find that the coefficients on EU_i,t_*ACCR_i,t_ are −0.070 and − 0.007 in columns (3) and (4), respectively. All of these coefficients on EU_i,t_*ACCR_i,t_ are negative and significant, which indicates that environmental uncertainty exacerbates the accrual anomaly in the Chinese capital market. The empirical results all support the hypothesis proposed in the paper with different methods of measuring environmental uncertainty. Therefore, we conclude that environmental uncertainty exacerbates the accrual anomaly indeed and the results are robust.

**Table 4:** Accrual anomaly test and the influence of environmental uncertainty on it.

Variable	(1)	(2)	(3)	(4)
	*CAR*			
	*Existence*	*EU = EU1*	*EU = EU2*	*EU = EU3*
*EU*		−0.002	0.006	−0.004***
		(−1.07)	(1.19)	(−10.48)
*ACCR*	−0.709***	−0.385***	−0.467**	−0.363*
	(−6.77)	(−3.98)	(−2.08)	(−1.81)
EU*ACCR		−0.131***	−0.070*	−0.007*
		(−2.76)	(−1.80)	(−1.73)
*CASH*	−0.502***	−0.382***	−0.603***	−0.440***
	(−4.90)	(−3.97)	(−5.54)	(−5.03)
*SIZE*	−0.045***	−0.044***	−0.047***	−0.045***
	(−14.10)	(−13.83)	(−13.75)	(−14.20)
*LEVEL*	0.041**	0.051***	0.040**	0.043**
	(2.19)	(2.70)	(1.98)	(2.28)
*EP*	0.943***	0.871***	1.088***	0.922***
	(7.03)	(6.62)	(7.16)	(7.07)
*BATA*	−0.058***	−0.058***	−0.065***	−0.057***
	(−4.23)	(−4.18)	(−4.46)	(−4.15)
*_CONS*	1.295***	1.259***	1.318***	1.435***
	(16.76)	(16.47)	(15.17)	(18.28)
*N*	16281	16281	16281	16281
*ADJ- R2*	0.300	0.299	0.291	0.300
*F-value*	164.6	155.9	135.8	160.9

This table reports the results of estimating regressions Eqs. (4) and (5). All regressions include the base set of control variables (*SIZE*, *LEVEL*, *EP*, and *BATA*), and industry and year fixed effects. All variables are as defined in Table 1. *, **, *** Denote significance at the 10, 5, and 1% levels, respectively. The t statistic is listed in parentheses.

Tables 5–7 report the results of our further analysis (H2, H3 and H4). We conduct three group tests for subsamples based on the quality of internal control, ownership and media coverage, namely, Group 1, Group 2 and Group 3. The results are presented in Tables 5–7, respectively. From Table 5, we find that the coefficient on EU_i,t_*ACCR_i,t_ is −0.041 in column (1) while it is −0.218 and significant in column (2). This result is consistent with our expectation that the coefficient on EU_i,t_*ACCR_i,t_ is negative in state-owned enterprises and positive or non-significant in non-state-owned enterprises in the subsample estimation for Group 2. It shows that the impact of environmental uncertainty on the accrual anomaly in state-owned enterprises is more significant than that in non-state-owned enterprises. That is, the special character of state-owned enterprises aggravates the impact of environmental uncertainty on the accrual anomaly. So it supports the third hypothesis. Columns (3) to (6) report the results for the two additional measurements of environmental uncertainty, and the results are consistent with expectation and robust. From Table 6, we find that the coefficient on EU_i,t_*ACCR_i,t_ is −0.07 in column (1), while it is −0.146 in column (2). This is consistent with our expectation that the coefficient on EU_i,t_*ACCR_i,t_ is negative under poor internal control and is positive or non-significant under the better control in the subsample results for Group 1. Therefore, the impact of environmental uncertainty on the accrual anomaly is more significant in enterprises with poor internal control than for those with better internal control. This verifies the second hypothesis, namely, that the quality of internal control can alleviate the impact of environmental uncertainty on the accrual anomaly. Columns (3) to (6) report the results for the two additional measurements of environmental uncertainty. We find that all the coefficients on EU_i,t_*ACCR_i,t_ are completely consistent with previous estimations. Therefore, we conclude that the quality of internal control can alleviate the impact of environmental uncertainty on accrual anomaly and that the results are robust. From Table 7, we find that the coefficient on EU_i,t_*ACCR_i,t_ is −0.194 in column (1) while it is −0.061 but not significant in column (2). This result is consistent with our expectation that the coefficient on EU_i,t_*ACCR_i,t_ is negative in high media coverage samples and positive or non-significant in poor media coverage enterprises in the subsample estimation for Group 3. It shows that the impact of environmental uncertainty on the accrual anomaly in high media coverage enterprises is more significant. That is, the media coverage aggravates the impact of environmental uncertainty on the accrual anomaly. Columns (3) to (6) report the results for the two additional measurements of environmental uncertainty, and the results are consistent with expectation and robust, so hypothesis 4 is also proved. By grouping regression of Eq. 5, we verify the above assumptions and confirm the robustness of the conclusion.

**Table 5:** The relation between environmental uncertainty and accrual anomaly in Group 1.

Variable	*EU = EU1*		*EU = EU2*		*EU = EU3*	
	(1)	(2)	(3)	(4)	(5)	(6)
	*SOE = 0*	*SOE = 1*	*SOE = 0*	*SOE = 1*	*SOE = 0*	*SOE = 1*
*EU*	−0.001	−0.004	0.018***	−0.006	−0.005***	−0.004***
	(−0.40)	(−1.33)	(3.26)	(−0.77)	(−9.49)	(−5.84)
*ACCR*	−0.579***	−0.264**	−0.832***	−0.175	−0.837***	0.174
	(−4.23)	(−2.25)	(−3.04)	(−0.57)	(−3.30)	(0.59)
EU*ACCR	−0.041	−0.218***	0.016	−0.105*	0.002	−0.019***
	(−0.68)	(−2.97)	(0.33)	(−1.96)	(0.43)	(−3.27)
*CASH*	−0.564***	−0.232*	−0.627***	−0.390**	−0.619***	−0.395***
	(−4.27)	(−1.78)	(−4.62)	(−2.56)	(−4.52)	(−2.60)
*SIZE*	−0.044***	−0.033***	−0.045***	−0.036***	−0.045***	−0.036***
	(−11.81)	(−5.35)	(−11.90)	(−5.72)	(−11.82)	(−5.73)
*LEVEL*	0.051**	0.065**	0.041*	0.049	0.047**	0.046
	(2.15)	(2.01)	(1.76)	(1.51)	(2.02)	(1.43)
*EP*	0.993***	0.531**	0.997***	0.640***	1.017***	0.637***
	(6.31)	(2.39)	(6.32)	(2.72)	(6.47)	(2.70)
*BATA*	−0.079***	−0.025	−0.080***	−0.028	−0.079***	−0.027
	(−4.44)	(−1.22)	(−4.48)	(−1.33)	(−4.44)	(−1.30)
*_CONS*	1.243***	1.037***	1.175***	1.124***	1.420***	1.230***
	(13.64)	(7.76)	(12.32)	(8.09)	(14.88)	(9.08)
*N*	7831	8450	7831	8450	7831	8450
*ADJ- R2*	0.327	0.295	0.328	0.295	0.328	0.296

This table reports the results of estimating regressions Eq. (5), aiming to explore the relationship between the environmental uncertainty and accrual anomaly when the nature of property rights is different. All regressions include the base set of control variables (*SIZE*, *LEVEL*, *EP*, and *BATA*), and industry and year fixed effects. All variables are as defined in Table 1. *, **, *** Denote significance at the 10, 5, and 1% levels, respectively. The t statistic is listed in parentheses.

**Table 6:** The relation between environmental uncertainty and accrual anomaly in Group 2.

Variable	*EU = EU1*		*EU = EU2*		*EU = EU3*	
	(1)	(2)	(3)	(4)	(5)	(6)
	*High-IC*	*Low-IC*	*High-IC*	*Low-IC*	*High-IC*	*Low-IC*
*EU*	−0.003	−0.001	0.010	0.005	−0.007***	−0.003***
	(−1.06)	(−0.30)	(1.31)	(0.79)	(−11.86)	(−4.64)
*ACCR*	−0.402***	−0.704***	−0.833**	−0.157	−0.838***	0.048
	(−2.91)	(−4.93)	(−2.44)	(−0.58)	(−2.65)	(0.19)
EU*ACCR	−0.070	−0.146*	0.005	−0.123***	0.001	−0.018***
	(−1.14)	(−1.90)	(0.09)	(−2.61)	(0.10)	(−3.64)
*CASH*	−0.284*	−0.695***	−0.451**	−0.688***	−0.455**	−0.698***
	(−1.88)	(−5.72)	(−2.54)	(−5.68)	(−2.56)	(−5.77)
*SIZE*	−0.051***	−0.044***	−0.053***	−0.044***	−0.053***	−0.045***
	(−9.45)	(−10.58)	(−9.87)	(−10.68)	(−9.83)	(−10.78)
*LEVEL*	0.093***	0.007	0.083***	0.003	0.085***	0.005
	(3.41)	(0.25)	(3.02)	(0.12)	(3.09)	(0.18)
*EP*	0.702***	0.990***	0.798***	0.978***	0.805***	0.985***
	(3.27)	(6.14)	(3.55)	(6.06)	(3.58)	(6.10)
*BATA*	−0.016	−0.122***	−0.016	−0.123***	−0.016	−0.122***
	(−0.80)	(−6.76)	(−0.80)	(−6.83)	(−0.79)	(−6.78)
*_CONS*	1.389***	1.348***	1.404***	1.325***	1.705***	1.453***
	(11.61)	(12.31)	(11.38)	(11.59)	(14.20)	(12.99)
*N*	8142	8139	8142	8139	8142	8139
*ADJ- R2*	0.289	0.316	0.289	0.316	0.290	0.316
*F-value*	65.13	88.59	64.79	88.28	66.60	90.48

This table reports the results of estimating regressions Eq. (5), aiming to explore the relationship between the environmental uncertainty and accrual anomaly when the quality of internal control is different. All regressions include the base set of control variables (*SIZE*, *LEVEL*, *EP*, and *BATA*), and industry and year fixed effects. All variables are as defined in Table 1. *, **, *** Denote significance at the 10, 5, and 1% levels, respectively. The t statistic is listed in parentheses.

**Table 7:** The relation between environmental uncertainty and accrual anomaly in Group 3.

Variable	*EU = EU1*		*EU = EU2*		*EU = EU3*	
	(1)	(2)	(3)	(4)	(5)	(6)
	*High-MC*	*Low-MC*	*High-MC*	*Low-MC*	*High-MC*	*Low-MC*
*EU*	0.000	−0.003	0.014*	0.002	−0.002**	−0.007***
	(0.17)	(−1.47)	(1.94)	(0.26)	(−2.20)	(−16.69)
*ACCR*	−0.243*	−0.380***	0.347	−0.963***	0.183	−0.545**
	(−1.84)	(−2.64)	(1.12)	(−3.56)	(0.59)	(−2.15)
EU*ACCR	−0.194***	−0.061	−0.174***	0.047	−0.015**	−0.004
	(−2.76)	(−0.94)	(−3.13)	(1.05)	(−2.48)	(−0.84)
*CASH*	−0.345***	−0.293**	−0.386***	−0.506***	−0.395***	−0.505***
	(−2.75)	(−2.00)	(−2.88)	(−3.26)	(−2.94)	(−3.25)
*SIZE*	−0.035***	−0.050***	−0.035***	−0.052***	−0.035***	−0.052***
	(−7.72)	(−9.12)	(−7.79)	(−9.71)	(−7.82)	(−9.69)
*LEVEL*	0.028	0.078***	0.022	0.064**	0.024	0.064**
	(0.99)	(2.93)	(0.79)	(2.38)	(0.85)	(2.37)
*EP*	0.916***	0.551***	0.926***	0.696***	0.942***	0.698***
	(5.42)	(2.76)	(5.37)	(3.42)	(5.46)	(3.41)
*BATA*	−0.082***	−0.011	−0.086***	−0.013	−0.083***	−0.012
	(−4.39)	(−0.56)	(−4.58)	(−0.61)	(−4.46)	(−0.60)
*_CONS*	1.080***	1.272***	1.028***	1.334***	1.172***	1.587***
	(8.91)	(10.07)	(8.09)	(10.46)	(8.56)	(12.57)
*N*	8138	8143	8138	8143	8138	8143
*ADJ- R2*	0.290	0.332	0.291	0.332	0.290	0.333
*F-value*	61.63	95.81	62.12	95.52	63.63	97.75

This table reports the results of estimating regressions Eq. (5), aiming to explore the relationship between the environmental uncertainty and accrual anomaly when the media coverage is different. All regressions include the base set of control variables (*SIZE*, *LEVEL*, *EP*, and *BATA*), and industry and year fixed effects. All variables are as defined in Table 1. *, **, *** Denote significance at the 10, 5, and 1% levels, respectively. The t statistic is listed in parentheses.

### Robustness tests

**Figure 2:**
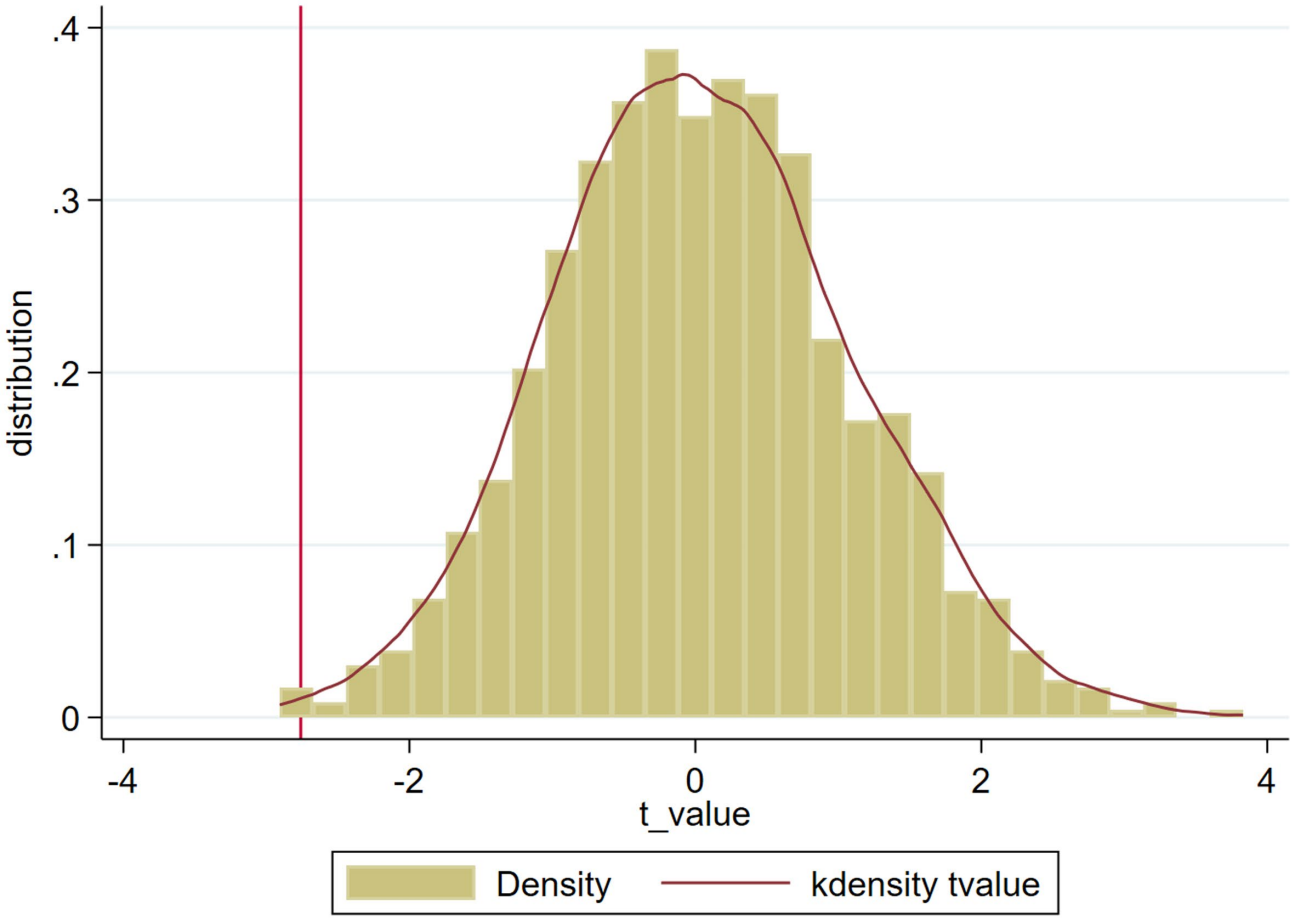
T-value distribution of placebo test. This figure depicts the distribution of T-values obtained from 1000 placebo tests. The vertical solid line represents the T-value of the standard regression cross-multiplication term, as shown in column (2) of Table 4.

We conduct multiple robustness tests by substituting variables, altering the sample selection criteria, performing a placebo test and measuring environmental uncertainty from multiple perspectives to support the results reported above. Here we only test the multiple robustness of the results of *EU1*. (i) Substituting measures. We replaced the consolidated daily market rate of return under the weighted average method of circulating market value (*R_p,d_*) with the consolidated daily market rate of return under the weighted average method of total market value (*R0_p,d_*) and recalculated the daily excess rate of return based on that market rate of return (*AR0_i,d_*), then adjusted to the required annual excess rate of return (*CAR0_i,t_*). Here, we take the new variable *CAR0_i,t \_* into account in the model and conduct a regression analysis. The results are shown in Table 8. (ii) Eliminate observations. The second robustness test is to reduce the sample size. Considering the influence of the subprime mortgage crisis in 2008, we delete observations from 2008, and the results are shown in Table 9. Meanwhile, from the descriptive statistics, we find that some firms have abnormal trading days. That is, a few of the enterprises in the sample have trades on only a few days, which may impact the messages on the company delivered to the market and influence our empirical results. Considering the feature of our sample, we eliminate the abnormal observations with fewer than 215 trading days, which represent 10% of the sample. After eliminating these firms, we conduct multiple regression analysis again, and the results are shown in Table 10. (iii) Placebo test. To rule out the possibility that the findings in this paper are the result of randomization, we construct a placebo treatment group based on the research of Chetty et al. (2009); that is, all environmental uncertainty values (*EU1*) are randomly assigned to all firms, and this randomization process is repeated 1000 times. Figure 2 shows that the T value of this randomized experiment is normally distributed around 0, and only a few random T values are less than the T value (−2.76) of the benchmark regression cross-multiplication term, so the assumptions of the placebo test are met. (iv) Measuring environmental uncertainty from multiple perspectives. As earlier in the article, to fully explore the impact of environmental uncertainty on the accrual anomaly, we build three indicators of environmental uncertainty from the micro, medium and macro perspectives. And all of the results are shown in the preceding table. The results of the robustness test are consistent with those in the preceding sections. Consequently, the conclusions above are robust and reliable.

**Table 8:** Robustness test: Substituting the measurement of *CAR.*

Variable	(1)	(2)	(3)	(4)	(5)	(6)	(7)
	*CAR0*						
	*Main*	*SOE = 0*	*SOE = 1*	*High-IC*	*Low-IC*	*High-MC*	*Low-MC*
*EU*	−0.002	0.000	−0.003	−0.001	−0.004	0.000	−0.003
	(−1.04)	(−0.26)	(−1.07)	(−0.39)	(−1.52)	(0.20)	(−1.47)
*ACCR*	−0.386***	−0.702***	−0.406***	−0.721***	−0.566***	−0.245*	−0.377***
	(−4.04)	(−4.91)	(−2.98)	(−4.64)	(−3.23)	(−1.86)	(−2.68)
EU*ACCR	−0.129***	−0.070	−0.143*	−0.005	−0.155**	−0.193***	−0.058
	(−2.73)	(−1.14)	(−1.86)	(−0.09)	(−1.99)	(−2.75)	(−0.89)
*CASH*	−0.379***	−0.693***	−0.281*	−0.611***	−0.403***	−0.347***	−0.283**
	(−3.97)	(−5.70)	(−1.87)	(−4.46)	(−2.65)	(−2.78)	(−1.96)
*SIZE*	−0.044***	−0.044***	−0.051***	−0.045***	−0.034***	−0.034***	−0.050***
	(−13.84)	(−10.64)	(−9.42)	(−11.79)	(−5.35)	(−7.72)	(−9.11)
*LEVEL*	0.051***	0.007	0.093***	0.048**	0.054*	0.028	0.078***
	(2.70)	(0.26)	(3.40)	(2.05)	(1.66)	(1.01)	(2.92)
*EP*	0.865***	0.987***	0.695***	1.012***	0.672***	0.907***	0.548***
	(6.59)	(6.13)	(3.24)	(6.42)	(2.88)	(5.37)	(2.76)
*BATA*	−0.058***	−0.122***	−0.016	−0.079***	−0.028	−0.083***	−0.011
	(−4.19)	(−6.79)	(−0.80)	(−4.41)	(−1.36)	(−4.41)	(−0.55)
*_CONS*	1.308***	1.403***	1.432***	1.300***	1.113***	1.128***	1.319***
	(17.11)	(12.82)	(11.97)	(14.16)	(8.25)	(9.29)	(10.43)
*N*	13367	8142	8139	7831	8450	8138	8143
*ADJ- R2*	0.304	0.282	0.307	0.322	0.285	0.279	0.325
*F-value*	130.8	66.28	87.67	-	87.08	58.48	98.14

This table reports the results of estimating regressions Eqs. (4) and (5), in which we replace *CAR* with *CAR0*. That is to say we use the total earnings as proxies for the accrual earnings, and then we take the new variable into the model and conduct regression. All regressions include the base set of control variables (*SIZE*, *LEVEL*, *EP*, and *BATA*), and industry and year fixed effects. *EU* is measured in *EU1*. All variables are as defined in Table 1. *, **, *** Denote significance at the 10, 5, and 1% levels, respectively. The t statistic is listed in parentheses.

**Table 9:** Robustness test: Eliminating samples in 2008.

Variable	(1)	(2)	(3)	(4)	(5)	(6)	(7)
	*CAR*						
	*Main*	*High-IC*	*Low-IC*	*SOE = 0*	*SOE = 1*	*High-MC*	*Low-MC*
*EU*	−0.002	0.000	−0.003	0.000	−0.004	0.001	−0.004*
	(−1.08)	(−0.25)	(−1.16)	(−0.19)	(−1.51)	(0.34)	(−1.80)
*ACCR*	−0.342***	−0.644***	−0.358***	−0.495***	−0.246**	−0.238*	−0.308**
	(−3.54)	(−4.35)	(−2.65)	(−3.43)	(−2.08)	(−1.74)	(−2.30)
EU*ACCR	−0.139***	−0.091	−0.142*	−0.048	−0.231***	−0.185**	−0.076
	(−2.85)	(−1.43)	(−1.80)	(−0.77)	(−3.05)	(−2.56)	(−1.14)
*CASH*	−0.382***	−0.693***	−0.272*	−0.549***	−0.240*	−0.343***	−0.282*
	(−3.89)	(−5.50)	(−1.78)	(−3.93)	(−1.81)	(−2.67)	(−1.94)
*SIZE*	−0.040***	−0.040***	−0.049***	−0.040***	−0.032***	−0.034***	−0.041***
	(−12.08)	(−8.99)	(−8.70)	(−10.02)	(−4.91)	(−7.31)	(−7.04)
*LEVEL*	0.038*	−0.012	0.087***	0.034	0.063*	0.029	0.058**
	(1.94)	(−0.42)	(3.06)	(1.39)	(1.89)	(0.98)	(2.06)
*EP*	0.880***	0.971***	0.692***	0.987***	0.565**	0.941***	0.524**
	(6.46)	(5.73)	(3.09)	(5.99)	(2.47)	(5.41)	(2.48)
*BATA*	−0.055***	−0.117***	−0.014	−0.079***	−0.020	−0.082***	−0.008
	(−3.94)	(−6.43)	(−0.70)	(−4.33)	(−1.07)	(−4.28)	(−0.39)
*_CONS*	1.187***	1.256***	1.344***	1.155***	1.001***	1.063***	1.098***
	(14.89)	(10.97)	(10.82)	(12.06)	(7.28)	(8.42)	(8.24)
*N*	15462	7732	7730	7566	7896	7718	7744
*ADJ- R2*	0.303	0.294	0.318	0.297	0.334	0.288	0.340

This table reports the results of estimating regressions Eqs. (4) and (5), in which we eliminate samples in 2008. All regressions include the base set of control variables (*SIZE*, *LEVEL*, *EP*, and *BATA*), and industry and year fixed effects. *EU* is measured in *EU1*. All variables are as defined in Table 1. *, **, *** Denote significance at the 10, 5, and 1% levels, respectively. The t statistic is listed in parentheses.

**Table 10:** Robustness test: Eliminating the abnormal samples.

Variable	(1)	(2)	(3)	(4)	(5)	(6)	(7)
	*CAR*						
	*Main*	*High-IC*	*Low-IC*	*SOE = 0*	*SOE = 1*	*High-MC*	*Low-MC*
*EU*	−0.001	−0.001	−0.001	0.000	−0.003	0.002	−0.002
	(−0.40)	(−0.29)	(−0.31)	(0.19)	(−1.17)	(0.67)	(−0.95)
*ACCR*	−0.431***	−0.704***	−0.526***	−0.769***	−0.632***	−0.336**	−0.375**
	(−4.15)	(−4.79)	(−3.06)	(−4.94)	(−3.45)	(−2.47)	(−2.54)
EU*ACCR	−0.121**	−0.069	−0.157*	0.024	−0.158**	−0.206***	−0.035
	(−2.47)	(−1.12)	(−1.78)	(0.38)	(−1.97)	(−2.80)	(−0.53)
*CASH*	−0.403***	−0.653***	−0.413**	−0.580***	−0.481***	−0.415***	−0.257*
	(−4.00)	(−5.27)	(−2.33)	(−4.22)	(−2.98)	(−3.22)	(−1.70)
*SIZE*	−0.046***	−0.047***	−0.056***	−0.047***	−0.034***	−0.037***	−0.052***
	(−13.78)	(−10.82)	(−8.79)	(−11.65)	(−5.11)	(−7.74)	(−9.20)
*LEVEL*	0.058***	0.013	0.103***	0.061**	0.047	0.026	0.093***
	(2.95)	(0.45)	(3.21)	(2.44)	(1.40)	(0.90)	(3.44)
*EP*	0.831***	0.948***	0.743***	0.982***	0.603**	0.874***	0.524***
	(6.25)	(5.76)	(3.14)	(6.19)	(2.54)	(5.12)	(2.62)
*BATA*	−0.074***	−0.126***	−0.048**	−0.088***	−0.046**	−0.103***	−0.018
	(−5.08)	(−6.63)	(−2.05)	(−4.63)	(−2.09)	(−5.20)	(−0.86)
*_CONS*	1.309***	1.396***	1.493***	1.276***	1.080***	1.151***	1.314***
	(16.51)	(12.95)	(10.67)	(13.39)	(7.76)	(9.09)	(10.27)
*N*	12692	7392	7392	6958	7826	7386	7398
*ADJ- R2*	0.308	0.297	0.302	0.334	0.292	0.293	0.334

This table reports the results of estimating regressions Eqs. (4) and (5), in which we eliminate the abnormal samples that trading days are less than 215 days, 10% of the sample. All regressions include the base set of control variables (*SIZE*, *LEVEL*, *EP*, and *BATA*), and industry and year fixed effects. *EU* is measured in *EU1*. All variables are as defined in Table 1. *, **, *** Denote significance at the 10, 5, and 1% levels, respectively. The t statistic is listed in parentheses.

## Further exploration

Many scholars have explored the cause of the accrual anomaly and offered four main hypotheses: the model-setting defect hypothesis, the investment growth hypothesis, the earnings persistence hypothesis and the limited arbitrage hypothesis. We further explore the mechanism of the impact of environmental uncertainty on the accrual anomaly, aiming to explain its causes in the Chinese capital market. Both information asymmetry and investor irrationality due to environmental uncertainty are the key factors affecting the accrual anomaly, so we explore the mechanism of environmental uncertainty affecting the accrual anomaly from the perspectives of behavioral finance in psychology.

### Information manipulation: The impact of accounting information quality

Present studies indicate that environmental uncertainty could aggravate the degree of information asymmetry between internal management and external stakeholders. When an enterprise is faced with high environmental uncertainty, management has a strong motivation to smooth earnings due to the considerations related to their own salary, career development and other personal factors (Habib et al., 2010). An increase in environmental uncertainty could thus lead management to cover up their firms’ real performance by hiding bad news and exaggerating good news. The consequently deteriorated firm accounting information does not properly and effectively reflect the enterprise-specific operations situation (Richardson et al., 2006). As the most important information appraising the performance of listed companies, accounting information is the major basis on which investors make their evaluations and is also an important reference for securities regulators. It is well known that investors evaluate the internal value of a company based on accounting information. Therefore, the quality of accounting data is not only an important basis for investor decision-making but also a key factor affecting investors’ ability to price accruals correctly (Kothari et al., 2006). When the information disclosed is of poor quality, the market cannot distinguish the persistence of accrual earnings and cash flow earnings, which aggravates the accrual anomaly in the capital market (Park et al., 2018). Therefore, we argue that the poor quality of accounting information caused by environmental uncertainty aggravates the accrual anomaly in the capital market.

### Management empire building: Impact of enterprise investment growth

Existing studies point out that environmental uncertainty increases the investment scale of enterprises from the perspective of cash holding (Baum et al., 2010) and may be especially likely to lead to increased investment in inefficient, low-growth, or “vanity” projects to meet personal self-interest or evade risks. Environmental uncertainty not only increases the information asymmetry between enterprises and investors but also disperses the energies of external regulators and internal audit departments. Thus, uncertainty complicates the prediction and supervision of management behavior, providing favorable conditions for management to hide decision-making mistakes and cover up operational defects (Cormier et al., 2013). As a result, management has the opportunity and motivation to over-invest, seek private benefits and build empires. The investment growth of enterprises is determined by the equilibrium of market supply and demand and also follows the law of diminishing marginal returns of asset growth. Considering the notion of diminishing marginal returns, Fairfield et al. (2003) point out that accounting accruals are a part of the net operating assets of the enterprise and propose that the low persistence of accruals is caused by the diminishing marginal returns to investment brought by enterprise growth. This then causes the capital market anomaly. Consequently, we argue that environmental uncertainty exacerbates the accrual anomaly in the capital market through increasing enterprise investment growth.

### Investor irrationality: Impact of investor limited attention

The risk signal transmitted by environmental uncertainty will make investors feel a sense of panic over the uncontrollable future, and their strong risk perception level can easily induce anxiety, affecting investment decisions. Under the uncertainty of the environment, the public shows an extreme desire for information, which is contrary to the behavior of the management to deliberately conceal information. This kind of contradiction will lead to the prevalence of gossip and rumors, resulting in artificial information asymmetry. The information asymmetry caused by environmental uncertainty increases the cost of investor information screening and diversifies the investors inattention, which makes them unable to respond quickly to risk information and triggers irrational behaviors (Bash and Alsaifi, 2019). Investors inattention is an important factor that triggers functional fixation on gross surplus, while the dispersion of investors limited attention due to environmental uncertainty exacerbates the accrual anomaly caused by irrational cognition (Shi and Zhang, 2012). Consequently, we argue that environmental uncertainty exacerbates the accrual anomaly in the stock market through distracting investors attention.

### Mechanism test

We use the mediating effects tests proposed by Hayes and Andrew (2009) to construct a panel mediation effect model to identify the mediating effect of environmental uncertainty on the accrual anomaly. And the equations are described in Eqs. (6) and (7):


(6) $$\mathrm{ME}D_{i,t}=\gamma_{0}+\gamma_{1}\;EU_{i,t}+\gamma_{2}\;\mathrm{CONTROL}S_{i,t}+\varepsilon_{i,t}$$

(7) $$\begin{array}{c} \begin{array}{l} CAR_{i,t+1}=\lambda_{0}+\lambda_{1}\;EU_{i,t}+\lambda_{2}\;ACCR_{i,t}+\lambda_{3}\;EU_{i,t}*ACCR_{i,t}\\ +\lambda_{4}\;\mathrm{ME}D_{i,t}+\lambda_{5}\mathrm{ME}D_{l_{i,t}}*ACCR_{i,t}+\lambda_{6}\;\mathrm{CONTROL}S_{i,t}+\varepsilon_{i,t} \end{array}\\ \; \end{array}$$

where *MED_i,t_* is the mediating variable. There are three different mediating variables, accounting information quality (*TRANS*), enterprise investment growth (*INVEST*) and investor attention (*IA*). Following prior studies (Ashbaugh-Skaife et al., 2008), accounting information quality (*TRANS*) is measured as information transparency, with the concrete steps of calculation as follows. We first use the Modified Jones Model to calculate the absolute value of discretionary accruals from year t-2 to year t and then we take the opposite of the number after calculating the sum of the absolute value above. A higher value of *TRANS* indicates better-quality accounting information. Investment growth (*INVEST*) is measured by the sum of net fixed assets and net inventory in year t scaled by total assets in year t (Lyandres et al., 2008). Drawing on existing research (Loh, 2010), we use the average turnover rate of the 30-day trading days before the announcement to measure investor attention (*IA*). A higher value indicates a higher investor attention or a light investor inattention.

Referring to a prior study (Wen and Fan, 2015; Guo et al., 2022), we use Eqs. (5–7) to test the mediating effect. Equation (5) shows the direct relationship of environmental uncertainty on the accrual anomaly, and *β_1_* is the total effect of environmental uncertainty on it. Equation (6) tests the effect of the independent variable environmental uncertainty on the mediating variable *MED* (*TRANS*, *INVEST*, and *IA*), and *γ_1_* is the effect of environmental uncertainty on the mediating variable. Equation (7) is developed for the direct effect and mediating role of environmental uncertainty and the accrual anomaly. And the coefficient *λ_5_* is the effect of the mediating variable *MED* on the accrual anomaly after controlling for the effect of environmental uncertainty, and the coefficient *λ_3_* is the direct effect of environmental uncertainty on the accrual anomaly after controlling for the effect of *MED*. The results are reported in Table 11.

**Table 11:** The mediating effect tests.

Variable	(1)	(2)	(3)	(4)	(5)	(6)	(7)
	Mode1(5)	Mode1(6)	Mode1(7)	Mode1(6)	Mode1(7)	Mode1(6)	Mode1(7)
	*CAR*	*MED = TRANS*		*MED=INVEST*		*MED=IA*	
*EU*	−0.002	−0.001***	−0.002	0.002**	−0.002	−0.000**	−0.002
	(−1.07)	(−2.77)	(−1.06)	(2.21)	(−1.12)	(−2.08)	(−1.35)
*ACCR*	−0.385***		−0.382***		−0.472***		−0.667***
	(−3.98)		(−3.99)		(−4.25)		(−4.90)
EU*ACCR	−0.131***		−0.126***		−0.137***		−0.084*
	(−2.76)		(−2.67)		(−2.72)		(−1.71)
*MED*			−0.006		0.063***		−1.198***
			(−0.13)		(2.86)		(−6.46)
MED*ACCR			1.303*		−0.216**		0.811
			(1.94)		(−2.13)		(0.28)
*CASH*	−0.382***		−0.383***		−0.441***		−0.550***
	(−3.97)		(−4.02)		(−4.20)		(−5.37)
*SIZE*	−0.044***	−0.006***	−0.043***	0.003*	−0.046***	−0.005***	−0.050***
	(−13.83)	(−6.47)	(−13.70)	(1.68)	(−13.66)	(−28.09)	(−14.90)
*LEVEL*	0.051***	0.046***	0.051***	0.107***	0.052**	0.007***	0.050***
	(2.70)	(8.27)	(2.66)	(10.92)	(2.50)	(6.77)	(2.63)
*EP*	0.871***	0.038	0.871***	0.013	0.998***	−0.009*	0.972***
	(6.62)	(1.40)	(6.63)	(0.23)	(6.69)	(−1.77)	(7.23)
*BETA*	−0.058***	−0.009***	−0.057***	−0.007	−0.063***	0.013***	−0.044***
	(−4.18)	(−3.43)	(−4.15)	(−1.31)	(−4.33)	(20.32)	(−3.11)
*_CONS*	1.259***	0.128***	1.253***	0.014	1.311***	0.117***	1.433***
	(16.47)	(5.97)	(16.36)	(0.37)	(15.86)	(28.61)	(17.37)
*N*	16281	16281	16281	16281	16281	16281	16281
*ADJ- R2*	0.299	0.0560	0.300	0.134	0.291	0.284	0.302
*F-value*	155.9	6.213	149.2	22.95	128.9	128.8	150.1

This table reports the results of estimating regressions Eqs. (5–7). All regressions include the base set of control variables (*SIZE*, *LEVEL*, *EP*, and *BATA*), and industry and year fixed effects. *EU* is measured in *EU1*. All variables are as defined in Table 1. *, **, *** Denote significance at the 10, 5, and 1% levels, respectively. The t statistic is listed in parentheses.

Whether we consider macro, medium-level, or micro environment uncertainty, the impact of uncertainty on firms is ultimately reflected in enterprise performance. Therefore, we use only one of the indicators, *EU1*, to test the mediating effect. When *MED_i,t_* is accounting information quality (*TRANS*), we find that the coefficient on EU_i,t_*ACCR_i,t_ in Eq. (5), reported in column (1), is −0.131, which is negative and significant. Next, the coefficient on *EU_i,t_* in Eq. (6), reported in column (2), is −0.001, which is negative and significant. Then, we observe that the coefficient on MED_i,t_*ACCR_i,t_ in Eq. (7) in column (3) is 1.303, which is positive and significant. For the test procedure, we need to judge whether the signs of γ_1_*λ_5_ and *λ_3_* are different. If they are the same, we conclude that environmental uncertainty aggravates the accrual anomaly by reducing the quality of accounting information and that accounting information quality plays a partial role in the mediating effect. When *MED_i,t_* is enterprise investment growth (*INVEST*), we find that the coefficient on EU_i,t_*ACCR_i,t_ in Eq. (5), reported in column (1), is −0.131, which is negative and significant. Next, the coefficient on *EU_i,t_* in Eq. (6), reported in column (4), is 0.002, which is positive and significant. Then, we observe that the coefficient on MED_i,t_*ACCR_i,t_ in Eq. (7) in column (5) is −0.216, which is negative and significant. We also find that the signs of γ_1_*λ_5_ and *λ_3_* are the same. We conclude that investment growth plays a partial mediating role when environmental uncertainty exacerbates the accrual anomaly. Lastly, when *MED_i,t_* is investor attention (*IA*), we find the coefficient on *EU_i,t_* in Eq. (6), reported in column (6), is −0.0002, which is negative and significant. And we observe that the coefficient on MED_i,t_*ACCR_i,t_ in Eq. (7) in column (7) is 0.811 which is positive but insignificant. For the test procedure, we need to take bootstrap test to check the null hypothesis whether γ_1_*λ_5_ = 0. The result refuse the null hypothesis, that’s, γ_1_*λ_5_ is not equal to 0. And then we also find that the signs of γ_1_*λ_5_ and *λ_3_* are same. We also confirm that investor attention plays a partial mediating role when environmental uncertainty exacerbates the accrual anomaly.

In addition, we further validate our findings of mediating effects by using the most commonly used grouped regressions in international studies. In conducting the test of the mediating effect of accounting information quality (*TRANS*), we use the median of *TRANS* as the basis for grouping and perform group regressions. The group above the median is defined as *GTRANS = 1*, that is, the group with high accounting information quality; conversely, *GTRANS = 0*. When carrying out the mediating effect test of investment growth (*INVEST*), we take the median of *INVEST* as the grouping basis and perform group regression. Among them, the group higher than the median is defined as *GINVEST = 1*, that is, the group with high investment growth; conversely, *GINVEST = 0*. Meanwhile, when conducting the test of mediating effect of investor attention (*IA*), we use the median of *IA* as the basis for grouping and perform group regressions. The group above the median is defined as *GIA = 1*, that is, high investor attention group,; conversely, *GIA = 0*. The results are shown in Table 12. We find that the coefficient on EU_i,t_*ACCR_i,t_ is −0.149 in column (1), while it is −0.064 in column (2). This is consistent with our expectation that the coefficient*β_3_* is significant negative under high information quality and is non-significant under poor information quality group. Therefore, environmental uncertainty exacerbates the accrual anomaly by reducing the quality of accounting information. And we also get that *β_3_* is −0.078 in column (3), while it is −0.181 in column (4), which indicates that the effect of environmental uncertainty on anomalies is more significant in the sample with low investment growth. Therefore, environmental uncertainty also exacerbates the accrual anomaly by increasing enterprise investment growth. Meanwhile, the coefficient*β_3_* is −0.119 and significant negative in column (5), while it is −0.062 but non-significant in column (6), which indicates that the effect of environmental uncertainty on anomalies is more significant in the sample with high investor attention. Therefore, environmental uncertainty also exacerbates the accrual anomaly by reducing investor attention.

**Table 12:** The mediating effect tests with group regression.

Variable	(1)	(2)	(3)	(4)	(5)	(6)
	*CAR*					
	*GTRANS = 1*	*GTRANS = 0*	*GINVEST = 1*	*GINVEST = 0*	*GIA = 1*	*GIA = 0*
*EU*	−0.001	−0.002	−0.003	−0.001	−0.001	−0.002
	(−0.38)	(−0.91)	(−1.23)	(−0.48)	(−0.39)	(−0.87)
*ACCR*	−0.303**	−0.553***	−0.422***	−0.364***	−0.597***	−0.482***
	(−2.56)	(−3.85)	(−3.27)	(−2.67)	(−5.06)	(−2.78)
EU*ACCR	−0.149**	−0.064	−0.078	−0.181***	−0.119*	−0.062
	(−2.05)	(−0.95)	(−1.18)	(−2.60)	(−1.77)	(−0.91)
*CASH*	−0.383***	−0.382***	−0.369***	−0.352**	−0.601***	−0.353**
	(−2.89)	(−2.81)	(−2.95)	(−2.51)	(−4.87)	(−2.39)
*SIZE*	−0.044***	−0.043***	−0.045***	−0.043***	−0.083***	−0.029***
	(−9.61)	(−9.18)	(−9.50)	(−10.29)	(−15.38)	(−6.80)
*LEVEL*	0.072**	0.030	0.060**	0.036	0.084***	0.042
	(2.57)	(1.11)	(2.08)	(1.41)	(3.27)	(1.52)
*EP*	0.698***	1.014***	0.934***	0.775***	1.066***	0.765***
	(3.68)	(5.72)	(4.83)	(4.54)	(5.30)	(4.58)
*BATA*	−0.060***	−0.057***	−0.034*	−0.083***	−0.053**	−0.026
	(−3.04)	(−2.92)	(−1.75)	(−4.35)	(−2.36)	(−1.42)
*_CONS*	1.301***	1.206***	1.258***	1.271***	2.115***	0.850***
	(11.69)	(11.13)	(11.34)	(12.14)	(17.92)	(7.25)
*N*	8141	8140	8141	8140	8141	8140
*ADJ- R2*	0.296	0.307	0.285	0.315	0.300	0.320
*F-value*	72.63	78.56	64.93	83.72	81.46	72.57

This table reports the results of estimating regressions Eq. (5). All regressions include the base set of control variables (*SIZE*, *LEVEL*, *EP*, and *BATA*), and industry and year fixed effects. *EU* is measured in *EU1*. All variables are as defined in Variable definition table. *, **, *** Denote significance at the 10, 5, and 1% levels, respectively. The t statistic is listed in parentheses.

All in all, we argue that environmental uncertainty aggravates the accrual anomaly in the stock market by reducing the quality of accounting information, increasing enterprise investment growth or aggravating investor inattention. All of them act as the partial mediator in this relationship.

## Conclusion

Consistent with prior work, we prove that there is an accrual anomaly in the Chinese stock market and explore the impact of environmental uncertainty on the anomaly. We find that environmental uncertainty exacerbates it. Next, we show that the impact of environmental uncertainty on the accrual anomaly is more significant in enterprises with poor internal control than in those with higher-quality internal control. And the correlation between the two is stronger in state-owned than the results in non-state-owned enterprises. Meanwhile, we also find that the relation between them is more significant in more media coverage samples. Furthermore, we find that the quality of accounting information, investment growth and investor attention all play the mediating role in the effect of environmental uncertainty on the accrual anomaly. This is, environmental uncertainty aggravates the accrual anomaly in the Chinese stock market by reducing the quality of accounting information, increasing corporate investment growth or reducing investor attention.

Our conclusions indicate that there is indeed an accrual anomaly in the Chinese stock market and that environmental uncertainty is an important factor affecting the anomaly. Better-quality internal control can significantly alleviate the impact of environmental uncertainty on accrual anomaly, which shows that the quality of internal control reflects that of corporate governance overall. That is, strong internal control can not only alleviate the agency problem but also reduce the degree of information asymmetry. Based on these findings, internal control is a key factor in alleviating the accrual anomaly. Moreover, we find that the special nature of state-owned enterprises aggravates the impact of environmental uncertainty on the accrual anomaly, which shows even if state-owned enterprises face environmental uncertainty, their management still desalinates risks and over invests due to government protection; that is, these firms with resource advantages aggravate the impact of environmental uncertainty on the accrual anomaly. Meanwhile, we also point that media coverage is one of the external factors that exacerbate the impact of environmental uncertainty on anomalies. It indicates that the media has not effectively played the role of external supervision and information intermediary, but excessive media coverage create noise that interferes with the investor cognition. Furthermore, we analyze the transmission mechanism (mediating effect) on the accrual anomaly of accounting information quality, enterprise investment growth and investor attention, which provides a new perspective on how to alleviate the accrual anomaly.

The policy implications of our study are reflected in the following four areas. First, enterprises do not exist independently of the external environment, so they should focus on improving the quality of internal control and enhancing their ability to stay below risk to cope with environmental uncertainty. Second, regulators should not only strengthen their supervision of the quality of information disclosure but also monitor the behavior of executives on multiple dimensions, such as surplus management, empire building and so on. Reducing the self-interest behavior of management is an important guarantee to deal with uncertainty risk and improve the efficiency of capital market pricing. Third, investors should filter information from multiple perspectives to reduce irrational behavior in decision-making, which is fundamentally one way to mitigate capital market anomalies. It’s necessary to improve their personal professionalism, build a diversified risk decision-making mindset, and incorporate environmental uncertainty into risk decision-making models to improve decision-making efficiency. Last but not least, as a medium for information transmission, media workers should always keep their original intention of spreading information objectively, fairly and truthfully, but the industry atmosphere has led some employees to abandon their professional ethics. So it is important to regulate the code of conduct of media worker to improve the credibility of media coverage.

## Data availability statement

The original contributions presented in the study are included in the article/supplementary material, further inquiries can be directed to the corresponding author.

## Author contributions

SW provided preliminary ideas about the research and revised the manuscript. SW and SH designed the structure. SH performed the statistical analysis and wrote the first draft of the manuscript. All authors contributed to the article and approved the submitted version.

## Funding

This work was supported by the National Natural Science Foundation of China (71862029) and the Key Research Center for Social Sciences of Committee of Education of Xinjiang, China (XJ2021G095). We gratefully acknowledge financial support from these funds.

## Conflict of interest

The authors declare that the research was conducted in the absence of any commercial or financial relationships that could be construed as a potential conflict of interest.

## Publisher’s note

All claims expressed in this article are solely those of the authors and do not necessarily represent those of their affiliated organizations, or those of the publisher, the editors and the reviewers. Any product that may be evaluated in this article, or claim that may be made by its manufacturer, is not guaranteed or endorsed by the publisher.
